# Exploring cortical excitability modulation to promote cognitive resilience in aging: an rTMS study protocol

**DOI:** 10.3389/fnhum.2026.1743236

**Published:** 2026-02-24

**Authors:** Chiara Di Fazio, Eugenio Scaliti, Mario Stanziano, Anna Nigri, Greta Demichelis, Marco Tamietto, Sara Palermo

**Affiliations:** 1 Department of Psychology, University of Turin, Turin, Italy; 2 International School of Advanced Studies, University of Camerino, Camerino, Italy; 3 Human Science and Technologies, University of Turin, Torino, Italy; 4 Department of Management “Valter Cantino”, University of Turin, Torino, Italy; 5 Neuroradiology Unit, Diagnostic and Technology Department, Fondazione Istituto di Ricovero e Cura a Carattere Scientifico (IRCCS) Istituto Neurologico Carlo Besta, Milan, Italy; 6 ALS Centre, “Rita Levi Montalcini” Department of Neuroscience, University of Turin, Turin, Italy; 7 Department of Medical and Clinical Psychology, Tilburg University, Tilburg, Netherlands; 8 Neuroscience Institute of Turin (NIT), Turin, Italy

**Keywords:** brain aging, cognitive resilience, cortical excitability, dorsolateral prefrontal cortex, high-frequency repetitive TMS, neuroplasticity, quality of life, repetitive transcranial magnetic stimulation

## Abstract

Promoting cognitive resilience in aging is essential for preserving autonomy and quality of life. Non-invasive brain stimulation techniques, such as repetitive transcranial magnetic stimulation (rTMS), have shown promise in enhancing neuroplasticity and cognitive functioning in older adults. This study protocol outlines the methodological framework for an investigation designed to examine whether high-frequency rTMS applied over the left dorsolateral prefrontal cortex (DLPFC) can modulate cortical excitability (CE) and characterize changes in cognitive and emotional functioning in healthy older individuals. The protocol provides detailed descriptions of stimulation parameters, safety monitoring procedures, and assessment tools. Cortical excitability will be measured using transcranial magnetic stimulation–derived motor-evoked potentials (MEPs), while cognitive and emotional outcomes will be assessed with a comprehensive neuropsychological battery. A preliminary feasibility phase with four participants was conducted to refine procedures and assess tolerability, safety, and data acquisition reliability. The study aims to determine the feasibility and signal characterization of cortical excitability modulation within a crossover framework and to explore the potential relationship between CE modulation and behavioral outcomes. Observations from this pilot phase will inform procedural refinement and the design of a larger ongoing trial.

## 1 Introduction

As the global population ages, understanding and addressing age-related changes in the brain, particularly cognitive decline and mood disorders, has become increasingly crucial (Amanzio and Palermo, 2021; Di Fazio et al., 2024; Ferreira et al., 2016; Amanzio et al., 2021; Stern, 2012). Promoting brain health is essential not just for extending life, but also for improving quality of life and maintaining cognitive function and resilience in older age (Amanzio and Palermo, 2021; Morese and Palermo, 2022; Palermo and IntechOpen, 2021). Cognitive resilience, defined as the mind’s capacity to adapt and maintain functionality in the face of stressors and changes, is a key aspect of protection against decline with aging (Joshi and Galvin, 2022; de Vries et al., 2024; Stern et al., 2023). Research on cognitive resilience offers valuable information regarding how older adults are able to sustain independence and general quality of life (Prince et al., 2024).

Identifying reliable biomarkers to assess cognitive resilience and predict cognitive decline is a significant priority in aging research. Cortical excitability (CE), which reflects the tendency of the cerebral cortex to generate electrical activity in response to stimuli, holds great potential as an important and less invasive biomarker for the comprehension of cognitive health (Menardi et al., 2018; Jannati et al., 2023). CE is related to the brain’s adaptive capacity and reflects the balance of excitatory and inhibitory processes. While CE generally declines with age, studies suggest that the relationship between CE and cognitive performance can be complex, potentially following an inverted-U hypothesis where both excessive and diminished excitability can impair function (Menardi et al., 2018). Lower CE has been observed in older adults with preserved robust cognitive performance compared to those with less cognitive robustness (Menardi et al., 2018; Palermo et al., 2025). Measuring CE non-invasively can provide insights into neuronal network communication and how aging affects different networks (Cespón et al., 2022; Berns et al., 2020).

Non-invasive brain stimulation (NIBS) techniques, such as transcranial magnetic stimulation (TMS), have emerged as tools to assess and potentially modulate CE, offering a promising approach to understanding and influencing cognitive aging (Buch et al., 2011; Rossi et al., 2009; Rossini and Rossi, 2007; Di Fazio et al., 2025a; Di Fazio et al., 2025b). Repetitive transcranial magnetic stimulation (rTMS) is a type of NIBS that employs magnetic fields to modulate neuronal activity in targeted brain regions. rTMS can influence neurophysiological, affective, and cognitive brain functions (Buch et al., 2011; Pascual-Leone and Walsh, 2001; Santarnecchi et al., 2018; Silvanto et al., 2007). Prior evidence suggests that rTMS can modulate cortical plasticity, which is crucial for learning and memory, and may facilitate neurophysiological adaptations associated with cognitive resilience and cognitive reserve (Fitzsimmons et al., 2024; Zhang et al., 2025). rTMS has been studied for its effects on various cognitive functions, including executive functions, working memory, attention, and processing speed, which are often affected by age (Di Fazio et al., 2025a; Turriziani et al., 2012; Asgarinejad et al., 2024; Sharbafshaaer et al., 2023; Xu et al., 2024). The left dorsolateral prefrontal cortex (DLPFC) is a frequent target in rTMS applications due to its central role in executive functions, cognitive control, and mood regulation (Amanzio and Palermo, 2021; Asgarinejad et al., 2024; Cui et al., 2020; Taylor et al., 2019; Amanzio et al., 2011). Modulating the DLPFC with rTMS has shown potential for improving cognitive performance and mood in older adults (Palermo et al., 2025; Di Fazio et al., 2025b; Zhang et al., 2025; Cui et al., 2022).

Building on our previous theoretical framework (Palermo et al., 2025) and earlier single-case evidence (Di Fazio et al., 2025a), this protocol expands the research scope by introducing a group-based, cross-over design. The study will involve healthy older adults, carefully screened and enrolled in alternating active and sham high-frequency rTMS conditions. This within-subject approach enables direct intra-individual comparisons and improves the interpretability of potential longitudinal changes.

The primary aim of this protocol is to describe the methodological and analytical framework for investigating whether cortical excitability (CE), increasingly discussed as a potential biomarker of cognitive resilience and neuroplasticity, is sensitive to neuromodulatory intervention in aging. Neuromodulatory interventions in aging (Palermo et al., 2025; Menardi et al., 2022; Motta et al., 2018; Ferreri et al., 2021). Motor-evoked potentials (MEPs), elicited by single-pulse TMS over the primary motor cortex, are used as a well-established, non-invasive proxy for CE (Spampinato et al., 2023; Huerta and Volpe, 2009). By integrating neurophysiological and cognitive assessments, the study aims to characterize the feasibility and within-subject sensitivity of CE to rTMS-induced modulation, rather than to establish diagnostic validity or clinical efficacy. Importantly, CE is conceptualized here as a state-sensitive, *within-subject marker of neuroplastic responsiveness*, reflecting changes relative to each individual’s own baseline.

This protocol outlines the rationale, methodological framework, and technical implementation of a 12-week, high-frequency rTMS intervention targeting the left dorsolateral prefrontal cortex (DLPFC), a region critically involved in executive functions, cognitive control, and mood regulation (Myers-Schulz and Koenigs, 2012; Toepper et al., 2014). Active stimulation consists of 10-Hz rTMS delivered over the left DLPFC at an intensity of 120% of the individual resting motor threshold (rMT). Each session includes 800 pulses, administered in 20 trains of 40 pulses, and is conducted three times per week for a total of 36 sessions over 12 weeks. The present work is conceived as a protocol-focused contribution, aimed at describing and justifying the feasibility, assessment strategy, and methodological rationale of a crossover rTMS design in healthy aging. The study design includes pre- and post-intervention assessments at multiple time points to evaluate changes in cortical excitability (CE), indexed by motor-evoked potentials (MEPs), along with a comprehensive neuropsychological battery assessing processing speed, executive functioning, mood, and quality of life. By systematically examining the relationship between neuromodulation, cortical excitability, and cognitive outcomes, this protocol aims to examine the feasibility and within-subject sensitivity of CE as a potential biomarker of neuroplasticity and cognitive resilience in aging and to provide a methodological foundation for future large-scale investigations of rTMS-based interventions for healthy cognitive aging.

## 2 Study protocol presentation

This study protocol outlines a 12-week clinical trial with 36 rTMS sessions, administered three times per week, each lasting about one hour, with at least one rest day between consecutive sessions. The trial uses a cross-over design to reduce interindividual variability, with participants randomized to begin with either active or sham stimulation before switching to the other condition. The active and sham stimulation phases are administered consecutively, without a formal washout period between conditions, reflecting the feasibility-oriented nature of the pilot phase. The protocol includes a multimodal assessment framework to capture both functional and neurophysiological outcomes.

Specifically, participants will undergo:

- A comprehensive neuropsychological evaluation assessing cognitive performance, emotional state, and quality of life;
- Cortical excitability measurement using single-pulse TMS and motor-evoked potential (MEP) recordings;
- Repeated rTMS sessions conducted according to international safety and methodological guidelines.

### 2.1 Neuropsychological evaluation

Cognitive, emotional, and quality-of-life outcomes will be evaluated using a comprehensive neuropsychological battery administered at two key time points: baseline (T0) and post-intervention (T36). Intermediate time points (T1, T2) will be included only for MEP recordings during the pilot phase. To minimize practice effects at follow-up, parallel or alternate test versions will be used when available.

The battery is designed to assess global cognitive functioning, executive control, processing speed, attention, and emotional well-being, with particular focus on domains sensitive to aging and fronto-executive dysfunction.

Global cognition will be measured with the Addenbrooke’s Cognitive Examination–Revised (ACE-R) (Mioshi et al., 2006), which includes a Mini-Mental State Examination (MMSE) subscore (Folstein et al., 1975). Executive functioning and cognitive flexibility will be assessed using the Trail Making Test (TMT), administered in parallel forms (A and C), including analyses of completion time differences and the B/A ratio (Amodio et al., 2008; Reitan and Wolfson, 1995). Cognitive reserve will be estimated with the Cognitive Reserve Index Questionnaire (CRIq) (Nucci et al., 2012), providing an index of participants’ lifetime intellectual enrichment.

Mood symptoms will be evaluated with the Beck Depression Inventory–II (BDI-II) (Beck et al., 1996) and the Beck Anxiety Inventory (BAI) (Beck and Steer, 1993). Self-perceived health status and quality of life will be assessed using the 5-level EuroQol 5-Dimension (EQ-5D-5L) scale (Stolk et al., 2019; Feng et al., 2021) and the Fatigue Assessment Scale (FAS) (De Vries et al., 2004).

### 2.2 Cortical excitability assessment

Cortical excitability (CE) will be assessed using motor-evoked potentials (MEPs) elicited by single-pulse transcranial magnetic stimulation (spTMS) applied over the left primary motor cortex (M1). A figure-of-eight coil connected to a Magstim BiStim^2^ stimulator (Magstim Company, UK) will induce a posterior–anterior monophasic current. Participants will be seated comfortably in a dimly lit room, with the right arm supported on a padded armrest to minimize muscle tension.

The optimal stimulation site (“motor hotspot”) will be defined as the scalp position that produces the largest and most consistent MEPs in the right first dorsal interosseous (FDI) muscle. Surface electromyography (EMG) will be recorded using a Biopac MP − 160 system (Biopac, Goleta, CA, USA) with Ag/AgCl electrodes placed in a belly–tendon montage. EMG signals will be bandpass filtered (30–500 Hz), sampled at 2 kHz, and stored for offline analysis using custom MATLAB scripts (MathWorks, Natick, MA, USA). Trials contaminated by background EMG activity or artifacts will be visually inspected and excluded, ensuring at least 30 valid MEPs per time point.

The resting motor threshold (rMT) will be defined as the lowest stimulation intensity that elicits MEPs ≥50 μV in at least five out of ten consecutive trials. MEPs will then be recorded at 120% of each participant’s rMT, to ensure reliable suprathreshold responses and reduce variability, and peak-to-peak amplitudes will be averaged across valid trials for each assessment time point (Rossi et al., 2009; Battaglia et al., 2024a, b; Paracampo et al., 2018; Paracampo et al., 2017). These indices will serve as quantitative proxies of corticospinal excitability and will be analyzed alongside behavioral measures to examine potential rTMS-induced neuroplastic changes.

Participants will wear earplugs throughout stimulation, and continuous EMG monitoring will be maintained to ensure muscle relaxation. All procedures will follow the safety and methodological recommendations of the International Federation of Clinical Neurophysiology (IFCN) for the application of TMS.

### 2.3 rTMS protocol

**Figure 1:**
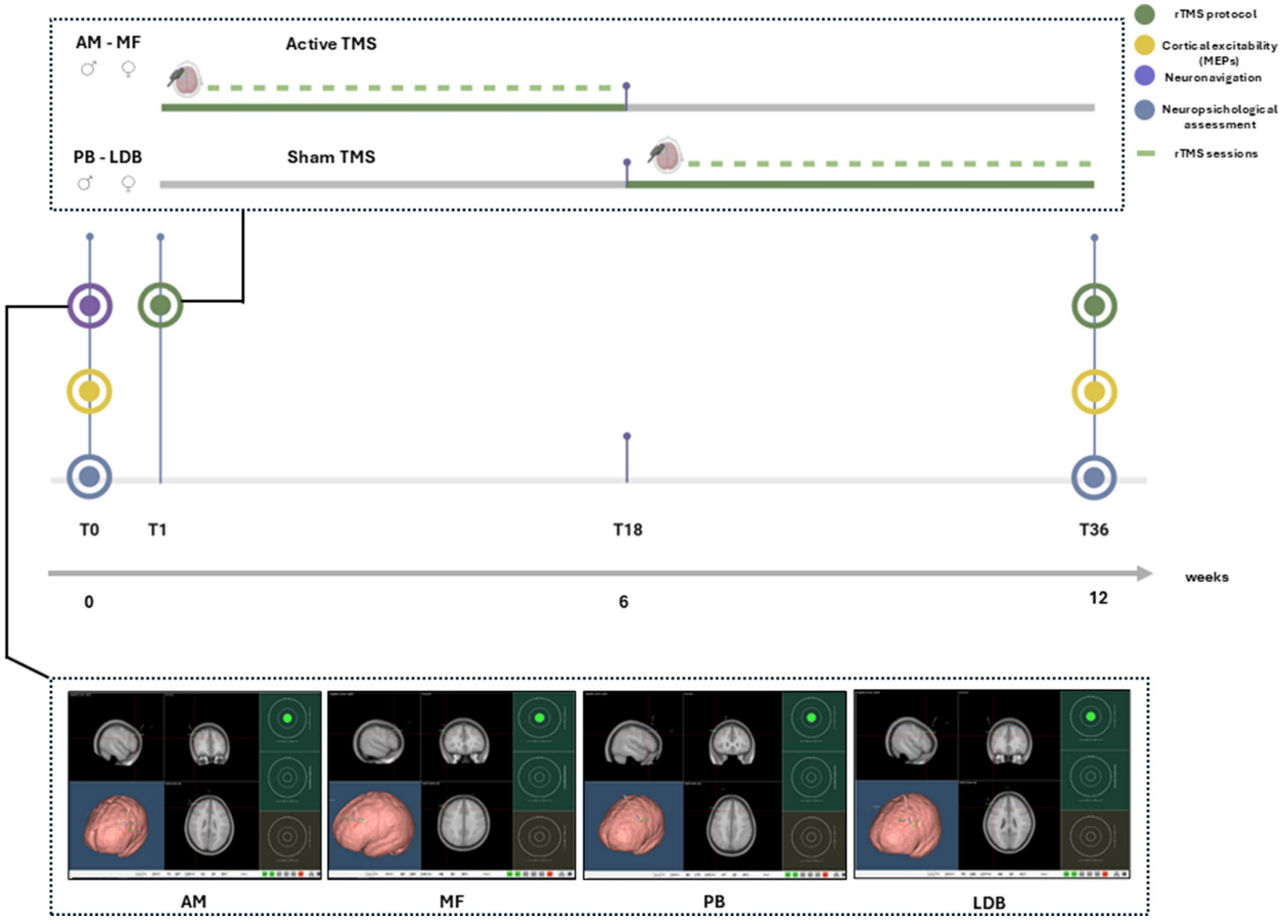
Workflow of the rTMS protocol. At baseline (T0), participants underwent neuropsychological assessment, cortical excitability (MEPs) recording, and a dedicated neuronavigation session to define the individualized stimulation target within the left DLPFC. Stimulation started immediately after baseline procedures and followed a cross-over design: two participants first received active rTMS, while the other two began with sham stimulation. Each intervention consisted of 36 sessions (three per week for 12 weeks). At the end of the stimulation phase (T36), all baseline measures were repeated.

The stimulation protocol will include a 12-week treatment phase consisting of 36 rTMS sessions, administered three times per week on alternate days. In the crossover design, participants will be randomly assigned to begin with either active or sham stimulation, followed by the alternate condition after completion of the first phase (Figure 1).

#### 2.3.1 Neuronavigation and targeting

To ensure accurate and reproducible targeting, neuronavigation will be performed using the SofTaxic Navigator system (Electro Medical Systems). Three cranial landmarks (nasion, inion, and preauricular points) will be digitized for each participant with a Polaris Vicra tracking system (Northern Digital, Canada). A customized MRI template will be adjusted to match individual cranial geometry, achieving an estimated spatial accuracy of approximately 5 mm. The subject-adjusted template will be obtained by warping a standard MRI template to individual cranial landmarks (nasion, inion, and preauricular points), without the use of individual structural MRI scans.

While this approach does not provide the same level of anatomical precision as individual MRI-based neuronavigation, previous validations indicate that landmark-based systems typically achieve spatial accuracy within the range of 5–8 mm. Potential sources of spatial error include inter-individual variability in cortical folding and scalp–cortex distance; however, this approach was selected as a pragmatic compromise between targeting accuracy, feasibility, and accessibility in non-clinical settings (Mert et al., 2013; Shamir et al., 2009; Lindseth et al., 2002).

The left dorsolateral prefrontal cortex (DLPFC) will be localized according to standard Talairach coordinates (*x* = −50, *y* = 30, *z* = 36), corresponding to the lateral portion of Brodmann area 9. The identified scalp position will be marked to ensure consistent coil placement across sessions (Palermo et al., 2025; Rossi et al., 2009; Di Fazio et al., 2025a,b). Coil orientation will be maintained at approximately 45° relative to the midsagittal plane to induce a posterior–anterior current flow.

#### 2.3.2 Stimulation parameters

Active stimulation will be delivered using a high-frequency 10 Hz rTMS protocol targeting the left DLPFC, a region widely associated with executive functioning and affective regulation. Each session will comprise 800 pulses, organized into 20 trains of 40 pulses each, separated by 50-s inter-train intervals, for a total duration of approximately 17 min (Di Fazio et al., 2025a; Zhang et al., 2025; Pan et al., 2023; Sabesan et al., 2015).

Stimulation will be applied using a figure-of-eight coil connected to an STM 9000 magnetic stimulator (ATES MEDICA Device, Verona, Italy). The stimulation intensity will be set at 120% of each participant’s resting motor threshold (rMT), determined at the beginning of each session following standard procedures (Palermo et al., 2025; Rossi et al., 2009; Di Fazio et al., 2025a,b). The rMT will be defined as the lowest intensity that elicits MEPs ≥50 μV in at least five out of 10 consecutive trials recorded from the right first dorsal interosseous (FDI) muscle.

Across participants, the average rMT is expected to be approximately 65% of maximum stimulator output (MSO), with a standard deviation of about ±2%.

#### 2.3.3 Sham stimulation and blinding

Sham stimulation will use the same coil, session duration, and neuronavigation-guided placement as the active condition. The coil will be tilted 90° relative to the scalp surface, eliminating effective magnetic field penetration while preserving the auditory and somatosensory sensations characteristic of active rTMS. which is widely used but may differ from active stimulation in somatosensory perception and carries a risk of partial unblinding. This method was selected to preserve similar auditory cues, session duration, and procedural characteristics while avoiding effective cortical stimulation. This approach is widely validated as a reliable sham condition (Poorganji et al., 2021; Grasin et al., 2019; Mennemeier et al., 2009).

In this protocol, potential unblinding effects will be monitored using post-session questionnaires assessing participants’ beliefs about the stimulation condition (Giustiniani et al., 2022). During all sessions, the experimenter administering the stimulation will be aware of the condition, while participants will remain blinded to active versus sham assignment.

### 2.4 Equipment and quality control

All TMS and EMG procedures will be performed using certified medical-grade equipment. Coil positioning and stimulation output will be recalibrated at the beginning of each session using the SofTaxic neuronavigation system to ensure spatial accuracy (error <5 mm). The same trained operator will conduct all stimulation sessions to minimize inter-operator variability, under the supervision of a clinician experienced in TMS procedures.

Equipment performance and safety compliance will be routinely verified according to manufacturer guidelines. Stimulation parameters – including intensity, pulse number, and coil position – will be automatically logged at each session to ensure full traceability. EMG data quality will be checked before acquisition for noise, signal stability, and electrode impedance; deviations from standard thresholds will be documented and corrected.

All raw EMG and stimulation data will be securely stored and backed up on encrypted drives to preserve data integrity. Participants’ comfort and safety will be continuously monitored during stimulation, and any adverse events or discomfort will be recorded according to established safety protocols. These procedures are designed to ensure methodological consistency, data reliability, and participant safety throughout the study.

### 2.5 Safety and tolerability

Safety and tolerability will be systematically monitored throughout the study. Before, during, and after each rTMS session, participants will be observed for potential adverse events or discomfort by a trained operator under medical supervision. Any side effects will be documented according to standardized procedures and classified by severity (mild, moderate, severe) and by their possible relationship to the stimulation protocol.

Post-treatment assessment of tolerability will be conducted using the standardized TMSens_Q questionnaire (Giustiniani et al., 2022), which evaluates perceived discomfort on a 0–4 scale and includes items assessing participants’ beliefs about the type of stimulation received (active or sham).

No major adverse events are anticipated. Based on previous evidence and pilot observations, mild and transient effects such as local scalp discomfort, muscle twitching, or mild tension-type headache may occur during the initial treatment sessions. These symptoms are typically rated 1/4 on the TMSens_Q scale and resolve spontaneously without medical intervention.

In the event that a participant experiences discomfort or intolerance at the planned stimulation intensity (120% rMT), stimulation intensity would be adjusted downward or the session would be temporarily interrupted or discontinued.

All rTMS sessions will be conducted in accordance with current safety and ethical guidelines of the International Federation of Clinical Neurophysiology (IFCN). In the event of any significant or unexpected adverse reaction, the session will be immediately interrupted, and appropriate medical evaluation will be performed. These procedures are designed to ensure participant safety, protocol compliance, and full adherence to international standards for non-invasive brain stimulation.

### 2.6 Data management and statistical plan

#### 2.6.1 Data management

All collected data (neuropsychological, neurophysiological, and demographic) will be securely stored on protected servers, accessible only to authorized members of the research team. Identifiable information will be kept separate from clinical data and coded using a unique alphanumeric identifier for each participant.

Raw data, like EMG recordings and MEP values, will be checked after each session to ensure integrity, completeness, and lack of artifacts. Regular backups will be performed on encrypted media.

Any modifications or corrections to the datasets will be tracked and documented according to internal audit procedures.

#### 2.6.2 Data analysis

Statistical analyses will be performed using standard software (e.g., SPSS, R, or MATLAB).

Given the exploratory nature of the study and the limited sample size, the main objective of the analysis will be descriptive and aimed at assessing the feasibility of the protocol.

Data will be summarized using descriptive statistics (mean, standard deviation, interquartile range), and within-subject effects will be calculated through comparisons between active and sham conditions.

- *Primary feasibility outcome:*
  The primary outcome of the feasibility phase is cortical excitability (CE), indexed by the average MEP amplitude, reflecting reliability, stability, and sensitivity of neurophysiological data acquisition. The feasibility/pilot phase is intended for procedural evaluation and signal characterization; therefore, pilot results will be reported descriptively and any within-subject modelling will be considered exploratory and hypothesis-generating, without confirmatory intent. Confirmatory analyses for the main ongoing trial will adopt a two-sided significance level of *α* = 0.05.
- *Secondary exploratory outcomes:*
  Secondary outcomes include cognitive performance (composite scores from neuropsychological tests assessing attention, executive functions, processing speed) and self-reported emotional well-being and quality of life (BDI-II, BAI, EQ-5D-5L, FAS). These measures are included for exploratory and descriptive purposes only, to assess assessment feasibility and variability, rather than efficacy.

For within-subject comparisons (active vs. sham; pre vs. post), non-parametric tests for paired samples (e.g., Wilcoxon or Friedman) or their parametric equivalents (repeated measures t-test, two-way ANOVA) will be used depending on data distribution. Effect sizes (Cohen’s d or r) will be calculated to estimate the magnitude of observed differences.

#### 2.6.3 Exploratory analyses

Correlations between changes in cortical excitability (ΔMEP) and changes in cognitive performance and psychometric scores will also be explored to evaluate the sensitivity of CE as a potential biomarker of neural plasticity and cognitive resilience.

#### 2.6.4 Data exclusion criteria

Data from sessions will be excluded if:

- The number of valid MEPs is fewer than 30;
- Uncorrectable EMG artifacts are present;
- The participant has not completed at least 80% of the scheduled sessions.

Confirmatory analyses for the main ongoing trial will use a two-sided significance level of *α* = 0.05. Analyses from the feasibility/pilot phase will be interpreted in an exploratory, non-confirmatory manner.

## 3 Case observations

Observations of individual cases can help verify procedural feasibility and methodological coherence of the protocol prior to larger-scale implementation. They allow us to evaluate tolerability, adherence, and measurement characteristics, and to illustrate inter-individual variability in a feasibility and exploratory framework. Given the small number of participants, these case descriptions are provided for context and protocol refinement rather than for drawing clinical or causal conclusions.

### 3.1 Participants

The study was approved by the Bioethical Committee of the University of Turin (Prot. n. 209329 of 08/04/2024) and conducted in accordance with institutional and international ethical standards. Participants were actively enrolled in educational courses at the University of the Third Age (UniTre) in Turin, and voluntarily sought access to the study in the context of subjective cognitive concerns or interest in maintaining cognitive health. All individuals provided written informed consent prior to any procedures. Their spontaneous engagement reflects both motivation toward lifelong learning and awareness of cognitive health.

The piloting sample included four healthy older adults (two males and two females), aged between 62 and 70 years (*M* = 66.5), all of whom were right-handed and had completed at least 13 years of formal education. All of them are subjects in typical cognitive aging and non-mild/major neurocognitive disorders in anamnesis.

Before participating in the study, each participant underwent a general medical examination by their GP. These examinations included a detailed review of medical history and confirmed that participants were in good health for their age at the time of enrollment in the study and had no clinical diagnoses or neurological/psychiatric conditions at the time of observation. The following individual case descriptions are reported to provide clinical and contextual information within a feasibility and exploratory framework, with the aim of illustrating inter-individual variability and participant characteristics.

This pilot study was instrumental for presenting and refining the study protocol; therefore, no formal sample size calculation was performed. The cross-over design allowed each participant to contribute data under both active and sham conditions, enhancing within-subject comparability despite the small N. Instead, the sample size was determined according to established recommendations for pilot studies—typically 10–12 participants per group to assess feasibility and estimate variability for planning larger trials (Julious, 2005; Whitehead et al., 2016). This approach ensured that the pilot fulfilled its primary purpose of testing procedures and informing the design of the comprehensive ongoing study.

- PB, a 64-year-old male with a degree in pharmacy, presented with subjective symptoms of demotivation, apathy, and anhedonia. His daily life had become increasingly isolated, and he expressed a loss of interest in formerly pleasurable activities. Despite these self-reported concerns, medical evaluation confirmed his good health and the absence of neurological or psychiatric conditions at study entry. He maintained functional independence and pursued technical hobbies at home. PB was motivated by the hope that the intervention could offer preventive or restorative cognitive support.
- AM, a 70-year-old male with an engineering background, reported occasional difficulties with attention and memory, particularly with names and places, although his daily functioning remained intact. He is intellectually and socially active, frequently attending lifelong learning courses and group activities. Medical evaluation at enrollment confirmed the absence of neurological or psychiatric disorders. His motivation to participate was driven by a desire to support his mental health during a period of increased emotional burden linked to caregiving responsibilities within his family.
- LDB, a 62-year-old female retired bank employee, reported a subjective progressive decline in concentration and difficulties in following conversations. She described recent life changes and high emotional stress due to complex family dynamics, including bereavement and caregiving for an elderly parent. At study entry, clinical assessment confirmed her good overall health, with no evidence of neurological or psychiatric disease. Although she remained functionally independent, she expressed a strong sense of fatigue, low mood, and social withdrawal. Her participation reflected a proactive attempt to regain psychological well-being and cognitive stability.
- MF, a 70-year-old female, requested access to treatment primarily for prevention and due to a reported loss of the ability to smell and taste. In March 2023, MF experienced a fall down the stairs, resulting in occipital head trauma with a focal contusion. Although she reported a transient loss of smell and taste following this mild traumatic brain injury, she had fully recovered by the time of study enrollment. Medical evaluation confirmed the absence of ongoing neurological sequelae, and MF performed within the normative range on all baseline cognitive and health assessments. Her participation was mainly motivated by prevention and the wish to maintain cognitive health over time.

At baseline (T0), all participants scored within the normal range on the neuropsychological battery (Table 1), mood scales, and MEP recordings. Descriptive comparisons of T0 scores showed no substantial inter-individual differences. Specifically, Mini-Mental State Examination (MMSE) scores ranged from 26.0 to 27.7 (*M* = 27.0), Addenbrooke’s Cognitive Examination-Revised (ACE-R) scores from 82.4 to 97.7 (*M* = 91.6), Beck Depression Inventory-II (BDI-II) scores from 2 to 10 (*M* = 6.3), Beck Anxiety Inventory (BAI) scores from 0 to 6 (*M* = 3.8), and Fatigue Assessment Scale (FAS) scores from 17 to 27 (*M* = 20.3). Baseline MEP amplitudes were also within physiologically valid ranges, varying between 0.15 and 0.88 mV across participants. These data confirm cognitive, emotional, and neurophysiological normality at study entry, providing a consistent reference for subsequent intervention-related changes.

**Table 1:** Outcomes of the neuropsychological assessment at baseline and at the follow-up.

AM		Baseline (T0)	Follow up (T36)	T36–T0 (∆)
*Quality of life assessment*				
EQ-5D-5L profile		11111	11111	
EQ-5D-5L index				
*Perceived health*				
FAS GLOBAL	<21	*17*	*16*	*−1*
*Mental*		*6*	*5*	*−1*
*Physical*		11	11	=
*Mood assessment*				
BDI-II	≤13	*2*	*1*	*−1*
BAI	≤7	0	2	+2
*Cognitive assessment*				
CRIq	>84	140	140	=
MMSE	≥23.8	27.7	27.7	=
ACE-R	>79	97.7	97.7	=
TMT A	≤87.32	*52*.*28*	*50.32*	*−1.96*
TMT B	≤269.14	*75*.*6*	*59.41*	*−16.19*

MF		Baseline (T0)	Follow up (T36)	T36–T0 (∆)
*Quality of life assessment*				
EQ-5D-5L profile		11111	11111	
EQ-5D-5L index				
*Perceived health*				
FAS GLOBAL	<21	*18*	*12*	*−6*
*Mental*		5	5	=
*Physical*		*13*	*7*	*−6*
*Mood assessment*				
BDI-II	≤13	*5*	*3*	*−2*
BAI	≤7	*3*	*1*	*−2*
*Cognitive assessment*				
CRIq	>84	147	147	=
MMSE	≥23.8	*26*	*27.2*	*+1.2*
ACE-R	>79	*82.4*	*93.2*	*+10.8*
TMT A	≤87.32	41.83	43.39	+1.56
TMT B	≤269.14	*71.4*	*50.70*	*−20.70*

PB		Baseline (T0)	Follow up (T36)	T36–T0 (∆)
*Quality of life assessment*				
EQ-5D-5L profile		21111	21111	
EQ-5D-5L index				
*Perceived Health*				
FAS GLOBAL	<21	*19*	*18*	*−1*
*Mental*		*8*	*7*	*−1*
*Physical*		11	11	=
*Mood assessment*				
BDI-II	≤13	10	10	=
BAI	≤7	6	6	=
*Cognitive assessment*				
CRIq	>84	133	133	=
MMSE	≥23.8	27.2	27.2	=
ACE-R	>79	89.2	89.2	=
TMT A	≤87.32	47.63	45.4	+2.23
TMT B	≤269.14	76.12	77.31	+1.19

LDB		Baseline (T0)	Follow up (T36)	T36–T0 (∆)
*Quality of life assessment*				
EQ-5D-5L profile		11134	11133	
EQ-5D-5L index				
*Perceived health*				
FAS GLOBAL	<21	*27*	*22*	*−5*
*Mental*		*14*	*12*	*−2*
*Physical*		*13*	*10*	*−3*
*Mood assessment*				
BDI-II	≤13	*8*	*7*	*−1*
BAI	≤7	6	6	=
*Cognitive assessment*				
CRIq	>84	125	125	=
MMSE	≥23.8	27.2	27.2	=
ACE-R	>79	97.2	97.2	=
TMT A	≤87.32	25.80	25.74	+0.06
TMT B	≤269.14	36.05	37.42	+1.37

Where cut-off scores exist, these are reported in the normal statistical direction. Changes between baseline and follow-up are shown as analysis of differences. *ACE-R*, Addenbrook’s Cognitive Examination Revised; *BAI*, Beck Anxiety Inventory; *BDI II*, Beck Depression Inventory version II; *CRIq*, Cognitive Reserve Index; *EQ-5D-5L*, 5-level EuroQol-5 Dimension; *FAS*, Fatigue Assessment Scale; *MMSE*, Mini Mental State Examination; *TMT*, Trail Making Test.

Values in italics indicate individual improvements observed from baseline (T0) to post-treatment (T36), including reductions in global and physical fatigue, improved mood and anxiety levels, and enhanced executive functioning, particularly on the Trail Making Test. These values are included for illustration and should not be interpreted as evidence of efficacy.

### 3.2 Methods

The rTMS protocol was applied to the described older adults who completed both the active and sham phases within the 12-week crossover design. Neuropsychological, cortical excitability, and self-reported measures were systematically collected at baseline and after the full intervention. Intermediate time points (T1, T2) were included only for MEP recordings during the pilot phase and were not part of the main behavioral assessment schedule. This pilot implementation confirmed the feasibility and safety of the protocol and provided critical insights to refine procedural parameters, optimize assessment timing, and ensure the robustness of the methodological framework for the upcoming larger-scale study.

Given the very small sample size (*N* = 4), no confirmatory group-level inference was planned or performed. Results are presented descriptively. Exploratory within-subject models were used only to characterize signal direction and within-individual variability in MEP-derived cortical excitability across time points, and should not be interpreted as evidence of efficacy, therapeutic benefit, or biomarker validation. The crossover design allows each participant to serve as their own control, thereby reducing inter-individual variability.

### 3.3 Piloting results

At the end of the active rTMS condition, both AM and MF showed marked within-individual changes compared to their own baseline values and compared to their results after sham stimulation. In MF, global fatigue decreased (Δ = −6), accompanied by a reduction in BDI-II (−2) and BAI scores (−2), indicating reduced depressive and anxiety symptoms. Cognitive measures also increased, with the MMSE rising from 26 to 27.2 and the ACE-R by +10.8 points (from 82.4 to 93.2), exceeding the clinical threshold. The executive function assessed with the TMT-B showed a reduction in completion time (Δ = −20.7 s), suggesting better task switching and cognitive flexibility. AM, who was already at a high baseline level, maintained his global cognition and mood but showed a reduction of 16.19 s on the TMT-B, suggesting a change in executive efficiency. His FAS score decreased only minimally (−1 point), remaining in the normal range, and no relevant changes were observed in perceived health or quality of life.

In contrast, neither PB nor LDB showed clinically meaningful changes at the end of sham treatment compared to their own baseline or to their results after active stimulation. ACE-R and MMSE scores were unchanged, and TMT performance showed only slight fluctuations: LDB showed a slight temporal deterioration (Δ = +1.37), while PB increased by +1.19 s on the TMT-B, indicating no reduction in completion time on this measure. Mood and anxiety symptoms remained stable, and fatigue decreased only slightly (PB: −1 point; LDB: −5 points), less pronounced than after active stimulation.

The cortical excitability data derived from the MEPs reflect this pattern. To assess changes in peak-to-peak measurement over time, a separate linear model was created for each subject using time point (T0, T1, T2) as a categorical predictor. Post-hoc comparisons were performed with Tukey-adjusted estimates. *p*-values are reported for transparency as part of exploratory within-subject modelling and should not be interpreted as confirmatory evidence. After active rTMS, the statistical models showed a time effect on peak-to-peak MEP amplitude in both AM and MF. For MF, the effect of time was statistically detectable (*F* (2, 43) = 28.50, *p* < 0.001). Post-hoc Tukey tests showed that mean MEP amplitudes decreased from 0.84 mV (T0) to 0.62 mV (T1) and 0.10 mV (T2), with pairwise contrasts indicating differences between T0–T2 (Δ = −0.74 mV, *p* < 0.001) and T1–T2 (Δ = −0.52 mV, *p* < 0.001), but not between T0–T1. A similar trend was observed for AM (*F* (2, 34) = 42.75, *p* < 0.001), with pairwise contrasts indicating differences from T0 to T1 and from T0 to T2, indicating early and sustained modulation of cortical reactivity. Post-hoc analyses revealed that mean MEP amplitudes decreased from 0.88 mV (T0) to 0.22 mV (T1) and 0.10 mV (T2). Pairwise contrasts indicated differences between T0–T1 (Δ = −0.65 mV, *p* < 0.001) and T0–T2 (Δ = −0.78 mV, *p* < 0.001), while the T1–T2 comparison was not supported by the exploratory contrasts at conventional thresholds (*p* = 0.40).

**Figure 2:**
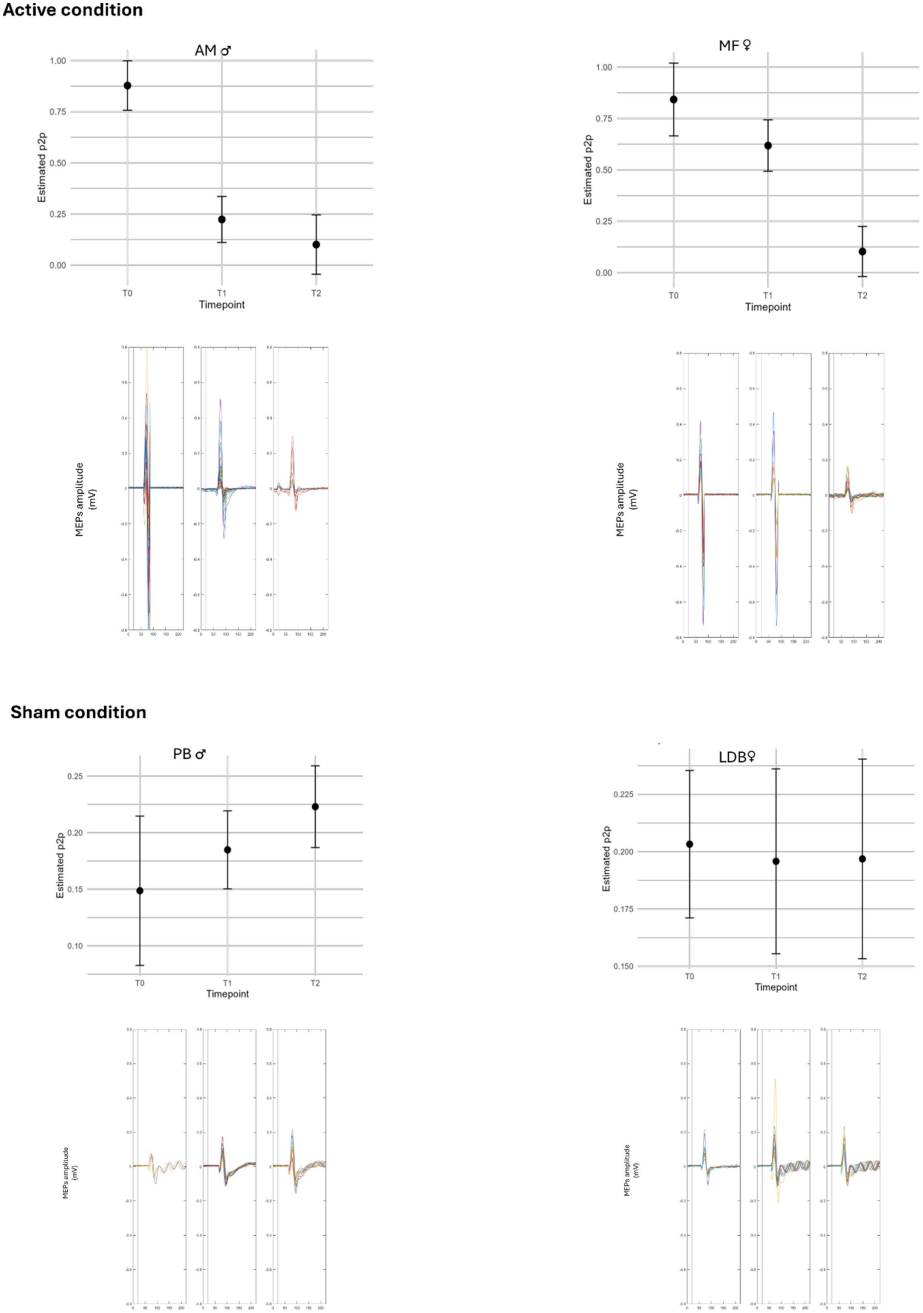
Assessment of cortical excitability measured through motor evoked potentials (MEPs) at baseline (T0) and post-intervention (T36) across the four participants following active and sham rTMS. Please note that values for cortical excitability in the sham group are displayed using a different y-axis scale to enhance visual clarity.

**Figure 3:**
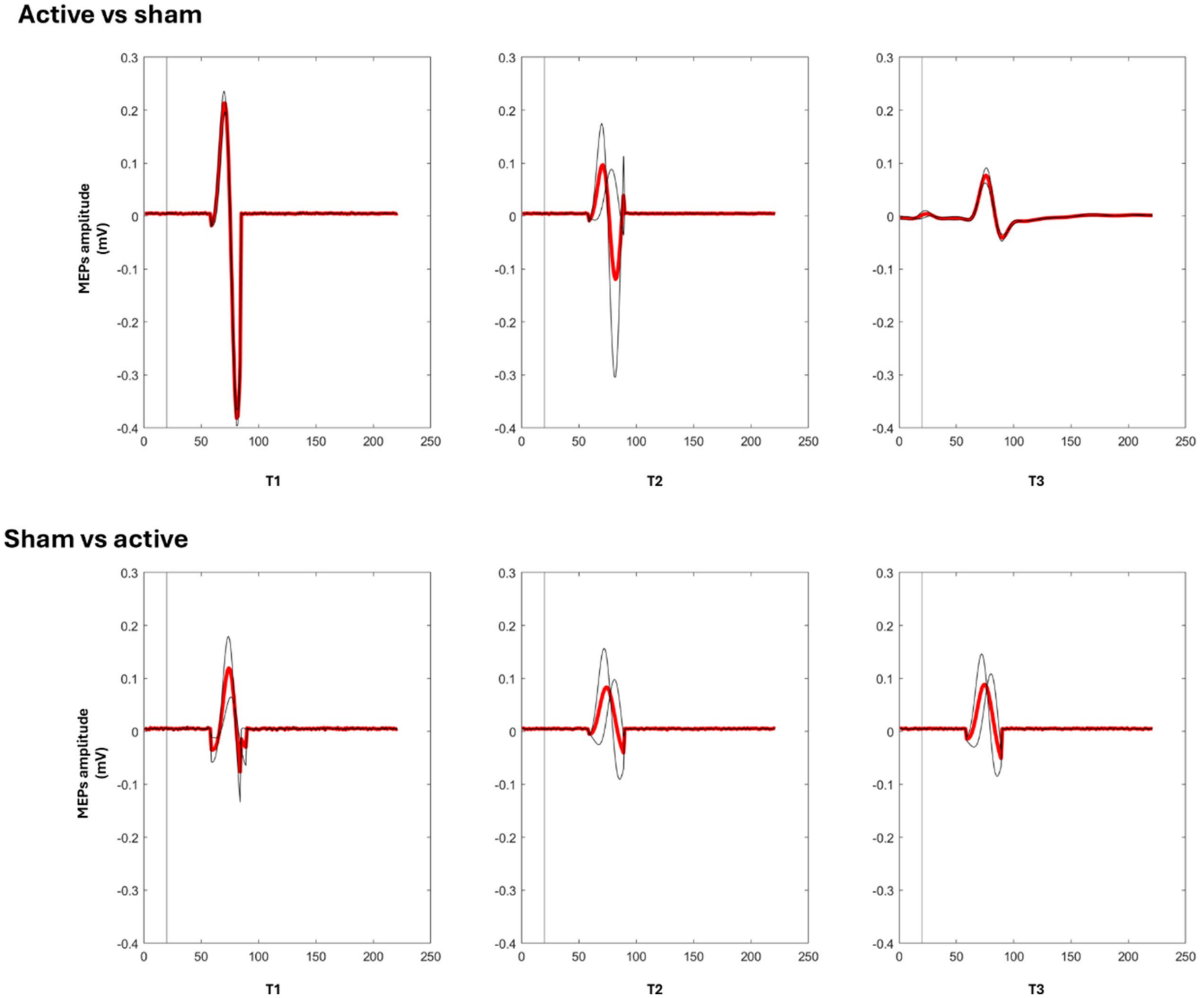
Mean cortical excitability (MEP amplitude) at baseline (T0) and post-intervention (T36) following active and sham rTMS. Data represent group-averaged values (±SEM) across participants.

In contrast, the exploratory models did not indicate a time effect at the end of the sham condition. For PB, the effect of time was not supported by the exploratory model (*F* (2, 21) = 2.26, *p* > 0.05); mean amplitudes were 0.15 mV (T0), 0.18 mV (T1), and 0.22 mV (T2), with all pairwise comparisons not supported at conventional thresholds (ps > 0.15). For LDB, the effect of time was also not supported by the exploratory model (*F* (2, 21) = 0.05, *p* > 0.05), with stable amplitudes across sessions (0.20 mV at all time points) and all pairwise comparisons not supported at conventional thresholds (ps > 0.95). This may suggest that repeated assessments alone were not sufficient to elicit neurophysiological changes and that the observed modulation of cortical excitability was more consistent with the active condition (Figures 2, 3).

## 4 Discussion

This work presents a study protocol designed to investigate whether cortical excitability, assessed through TMS-derived MEPs, may serve as a sensitive and reliable biomarker of neuroplastic changes following rTMS in older adults, and how such physiological modulation relates to neuropsychological outcomes. The accompanying piloting phase was instrumental in testing the feasibility, safety, and sensitivity of the procedures, while also providing preliminary observations in a small group of older adults. This within-subject design allowed each individual to serve as their own control, thereby increasing the internal validity of the findings despite the small sample size. Assessments were carried out at two timepoints: baseline (T0) and end of treatment (T36), to evaluate cumulative changes in cognitive functioning and cortical excitability observed following the full intervention. Although behavioral assessments were limited to T0 and T36, the crossover design allowed condition-specific comparisons by contrasting within-subject changes following active versus sham stimulation. This approach strengthened interpretability despite the reduced number of assessment timepoints. Moreover, the protocol was intentionally designed to capture cumulative, longer-term effects of repeated rTMS rather than acute modulation following single sessions; therefore, intermediate or immediate post-session assessments were not included to limit participant burden and repeated testing effects. By combining this crossover design with both neurophysiological and neuropsychological measures, the pilot data support the feasibility and methodological rigor of the protocol, while offering preliminary descriptive observations of measurable cortical and behavioral changes following high-frequency rTMS targeting the left DLPFC.

All participants underwent both active and sham stimulation within a cross-over design, and all comparisons were made relative to each participant’s own baseline and condition-specific outcomes. Within this protocol framework, the piloting results provide illustrative, hypothesis-generating within-subject trajectories. In the two participants who received active stimulation first, TMT-B completion times decreased from baseline to post-treatment, whereas changes following sham were smaller or inconsistent. These individual-level observations are not generalizable and may reflect practice, expectancy, or carryover effects; nevertheless, they show that the assessment battery can capture within-subject variability in executive performance across conditions, a key target of DLPFC stimulation. Indeed, the TMT-B is widely regarded as a sensitive index of attentional control, cognitive flexibility, and the efficiency of executive networks (Kortte et al., 2002; Varjacic et al., 2018; MacPherson et al., 2019; Linari et al., 2022; Seibert et al., 2025; Lazari et al., 2022; Chu et al., 2021; Xie et al., 2021; Zhang et al., 2022). In the context of this feasibility study, changes on this task are interpreted conservatively as descriptive indicators of protocol sensitivity rather than as evidence of cognitive enhancement.

From a neurocognitive perspective, contemporary models emphasize that higher-order cortical regions such as the lateral prefrontal cortex do not implement isolated cognitive operations, but instead shape adaptive control through top–down modulation of distributed networks (Martínez-Molina et al., 2024; Momi et al., 2021). Recent computational accounts suggest that prefrontal regions contribute to processes such as conflict expectation, contextual prediction, and flexible adjustment of control policies, dynamically influencing downstream sensorimotor and associative systems (Martínez-Molina et al., 2024; Momi et al., 2021). Within this framework, neuromodulation of the left DLPFC is unlikely to exert purely focal effects, but may bias the state of large-scale control networks involved in executive regulation.

Accordingly, changes in cortical excitability should not be interpreted as direct correlates of specific cognitive computations, but rather as state-dependent physiological reflections of broader network reconfiguration. In this context, MEP-derived cortical excitability is conceptualized as a global index of system-level responsiveness, sensitive to changes in distributed circuits engaged by prefrontal control (Martínez-Molina et al., 2024; Momi et al., 2021). Crucially, here we do not imply a focal or site-specific causal relationship between stimulation of the left DLPFC and motor cortex output. Instead, observed MEP modulation is interpreted as an indirect consequence of network-level dynamics elicited by prefrontal neuromodulation, in line with evidence that rTMS effects are more consistently expressed at the level of large-scale networks than at isolated cortical sites (Martínez-Molina et al., 2024; Momi et al., 2021; Menardi et al., 2022).

By contrast, outcomes related to self-reported fatigue and mood appeared more variable across conditions. While one participant in the active phase reported reductions in global fatigue and mood symptoms, similar decreases were also present in the sham condition, and another participant in the active phase showed only minimal changes. These mixed patterns highlight the inherent variability of subjective measures in small pilot samples and the need for cautious interpretation. Within this pilot, some changes appeared to co-occur with the active condition; however, practice, expectancy, and potential carryover effects cannot be excluded. Neurophysiological recordings also demonstrated that the protocol can acquire stable MEP measures and characterize within-individual changes across sessions in the piloting phase. In the two participants who received active stimulation first, MEP amplitudes varied across post-session time points (T0–T2) in the exploratory within-subject models; in contrast, MEP amplitudes were comparatively stable during sham. These descriptive patterns are compatible with stimulation-related modulation but cannot establish mechanism or efficacy in this feasibility sample (Xu et al., 2024; Avenanti et al., 2018; Turrini et al., 2023).

We therefore interpret them primarily as evidence of measurement feasibility and signal detectability, which supports the choice of MEP-derived cortical excitability as the primary physiological outcome in the planned study (Battaglia et al., 2024a; Paracampo et al., 2017; Avenanti et al., 2018; Borgomaneri et al., 2015; Borgomaneri et al., 2020; Borgomaneri et al., 2015; Borgomaneri et al., 2021). In one participant, the largest MEP change coincided with the largest descriptive cognitive change, suggesting a potential within-individual covariation that warrants systematic testing in adequately powered studies. In this protocol, cortical excitability is considered a state-sensitive physiological readout that may help stratify responsiveness; however, any association with behavioral outcomes remains a hypothesis and should be interpreted cautiously in the present pilot (Petitet et al., 2021; Yan et al., 2024).

A central aim of this protocol was to explore whether cortical excitability could function as a reliable biomarker of neuroplastic responsiveness within a structured study framework. Observations from the piloting phase suggest that CE captures both cortical responsiveness and meaningful inter-individual variability in potential for functional change. For example, MF, whose baseline profile included mild functional complaints, showed the largest modulation of CE alongside cognitive changes, whereas AM exhibited marked CE modulation with minimal behavioral variation. Although these observations are descriptive and not generalizable, they illustrate the protocol’s ability to detect differential responsiveness across individuals (Borgomaneri et al., 2020; Borgomaneri et al., 2015). Interpreting such variability requires consideration of the growing emphasis on personalization in non-invasive brain stimulation. Inter-individual differences in baseline cortical excitability, network integrity, cognitive reserve, and anatomical features are known to critically shape responsiveness to rTMS, particularly in aging populations (Figueroa-Vargas et al., 2024; Soleimani et al., 2025). In this protocol, a standardized stimulation approach was intentionally adopted to establish tolerability, procedural robustness, and baseline variability prior to introducing additional layers of individualization. Within this framework, heterogeneous or null responses are not interpreted as lack of neuromodulatory potential, but as reflecting meaningful individual differences that motivate subsequent personalized implementations (Figueroa-Vargas et al., 2024; Soleimani et al., 2025). Accordingly, low-cost personalization strategies (such as individualized frequency tuning or baseline excitability–informed dosing) represent natural extensions of the present design and will be systematically explored in future studies.

Although formal predictive analyses were not conducted due to the small sample size, qualitatively, participants with lower baseline cognitive performance or higher fatigue or mood symptoms showed greater CE and neuropsychological improvements, while high-functioning participants demonstrated robust CE enhancement with minimal behavioral change. These pilot observations suggest the protocol’s potential to detect baseline-dependent responsiveness (Figueroa-Vargas et al., 2024; Soleimani et al., 2025), a hypothesis that should be systematically tested in larger studies.

Taken together, these pilot findings indicate the feasibility of collecting MEP-derived cortical excitability and neuropsychological measures within the planned crossover schedule. The observed condition-related patterns are hypothesis-generating and consistent with the conceptual framework motivating cortical excitability as a candidate physiological marker for future confirmatory work, while underscoring the need for larger, adequately powered trials to test efficacy and longer-term impact (Di Fazio et al., 2025a; Sabesan et al., 2015; Hsu et al., 2016; Pavon et al., 2019; Miniussi et al., 2013; Cappon et al., 2022). Although the study involved only four participants, it was designed as an initial feasibility phase embedded within a structured longitudinal cross-over protocol. This methodological choice allowed us to test recruitment and adherence procedures, stimulation tolerability, and the acquisition of neurophysiological and neuropsychological measures under the planned schedule. The pilot observations should therefore be interpreted as descriptive and hypothesis-generating, and they primarily inform operational refinement, variance estimation, and the design of larger, adequately powered trials.

Several methodological limitations of the pilot phase should be acknowledged when interpreting these findings. The absence of a formal washout period between active and sham phases represents a methodological limitation of the pilot phase and may have introduced potential carryover effects. However, as the primary aim of this feasibility study was not to assess efficacy but to test procedural implementation, tolerability, and data acquisition reliability, this limitation does not undermine the methodological objectives of the protocol. In addition, potential sex-related differences in cortical excitability and responsiveness to rTMS could not be examined due to the very limited sample size and should be explicitly addressed in future studies with adequately powered and sex-balanced samples.

In conclusion, this manuscript presents a methodologically grounded rTMS protocol designed to examine cortical excitability modulation in aging within a crossover design. The feasibility phase indicates that the protocol allows reliable acquisition of neurophysiological and cognitive measures and captures within-individual variability across conditions. In this pilot, the clearest condition-related pattern was observed in MEP-derived cortical excitability; however, these observations are descriptive and may be influenced by carryover or expectancy effects, and they are not intended as evidence of efficacy. Within a network-level perspective, these observations motivate the use of cortical excitability as a system-level physiological outcome rather than as a direct marker of localized effects. The protocol is positioned to support future investigations explicitly testing neurocognitive and computational models of adaptive control and plasticity in aging, with larger trials required to confirm efficacy and long-term impact.
